# Spontaneous renal artery dissection: angioplasty with stent implantation in one-year follow-up

**DOI:** 10.31744/einstein_journal/2022RC6570

**Published:** 2022-03-29

**Authors:** Katia Pinheiro de Souza, Priscila Mina Falsarella, Felipe Nasser, Rodrigo Gobbo Garcia, Jairo Tabacow Hidal

**Affiliations:** 1 Hospital Israelita Albert Einstein São Paulo SP Brazil Hospital Israelita Albert Einstein, São Paulo, SP, Brazil.

**Keywords:** Renal artery, Dissection, Stents, Hypertension, renovascular, Angioplasty, Graft occlusion, vascular

## Abstract

Spontaneous renal artery dissection is an unusual and idiopathic condition in most cases. In young, mildly symptomatic patients, diagnosis may be difficult, frequently culminating in delay in treatment. This report presents the case of a 40-year-old male patient, with severe hypertension of sudden onset, and difficult management of oral medication. In etiological investigation, Echo-Doppler of renal arteries showed signs of hemodynamically relevant right renal artery stenosis. Arteriography showed presence of double-lumen and thrombus in the vessel lumen, indicating dissection. The proposed treatment was endovascular approach after failure of isolated medical treatment, option which included the aspiration of the thrombus by Penumbra System^®^ device and balloon angioplasty, followed by right renal artery stenting. Improvement of immediate sonographic control of peak systolic velocity and renal-aortic ratio was shown, with a consequent reduction of systemic arterial blood pressure and stabilization of renal function. Within the following year, the patient presented in-stent stenosis and was successfully treated with balloon angioplasty.

## INTRODUCTION

Renal artery stenosis is a pathological condition characterized by a narrowing of the artery, resulting in reduced blood flow to the organ.^(1)^ It triggers several compensatory mechanisms, among which the most known is the renin-angiotensin-aldosterone pressure regulation system, which increases blood pressure levels to compensate for the low blood flow to the kidney.

Spontaneous renal artery dissection is a rare cause of stenosis, and is defined as a dissection not related to trauma or previous interventions.^(2)^ The diagnosis may be difficult, considering its occurrence in young patients and the nonspecific symptoms, resulting in late treatment in many cases. Currently, there is still no consensus on which therapy is best indicated for these patients.^(1)^ Options range from a clinical treatment only, a minimally invasive endovascular approach, to open surgical treatment.

In this report, we aim to present a case of spontaneous renal artery dissection that progressed to malignant systemic arterial hypertension resistant to drug treatment only, which was managed with endovascular therapy.

## CASE REPORT

A 40-year-old male patient with no history of hypertension was admitted to the emergency room with a sudden rise in blood pressure (220mmHg x 170mmHg), associated with low back pain for one day. The patient reported practicing strenuous physical exercise on the day before the onset of symptoms. The initial laboratory evaluation showed an increase in serum creatinine levels (1.33), and ideal blood pressure control was not achieved with oral hypotensive agents and analgesia, and intravenous therapy with sodium nitroprusside was started to manage the hypertensive emergency.

**Figure 1:**
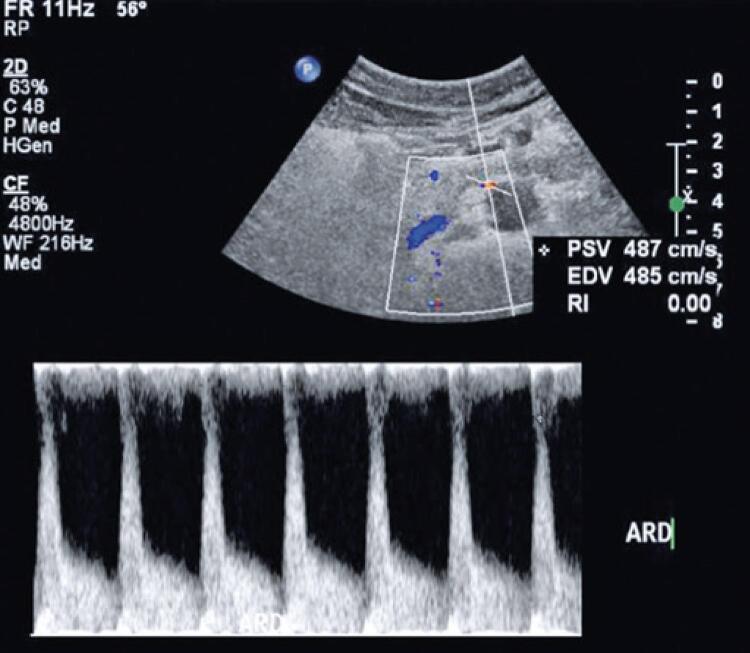
Diagnostic ultrasound showing increased peak systolic velocity in the right renal artery measured by Doppler

In view of the clinical course of the case, the hypothesis of secondary hypertension was raised, with consequent laboratory and imaging investigation. Renal artery Doppler ultrasonography showed signs of hemodynamically significant right renal artery stenosis, with peak velocities of up to 490cm/s (left renal artery values of 85cm/s) and marked reduction in intrarenal flow (Figure 1). In time, antiaggregation with clopidogrel was started.

**Figure 2:**
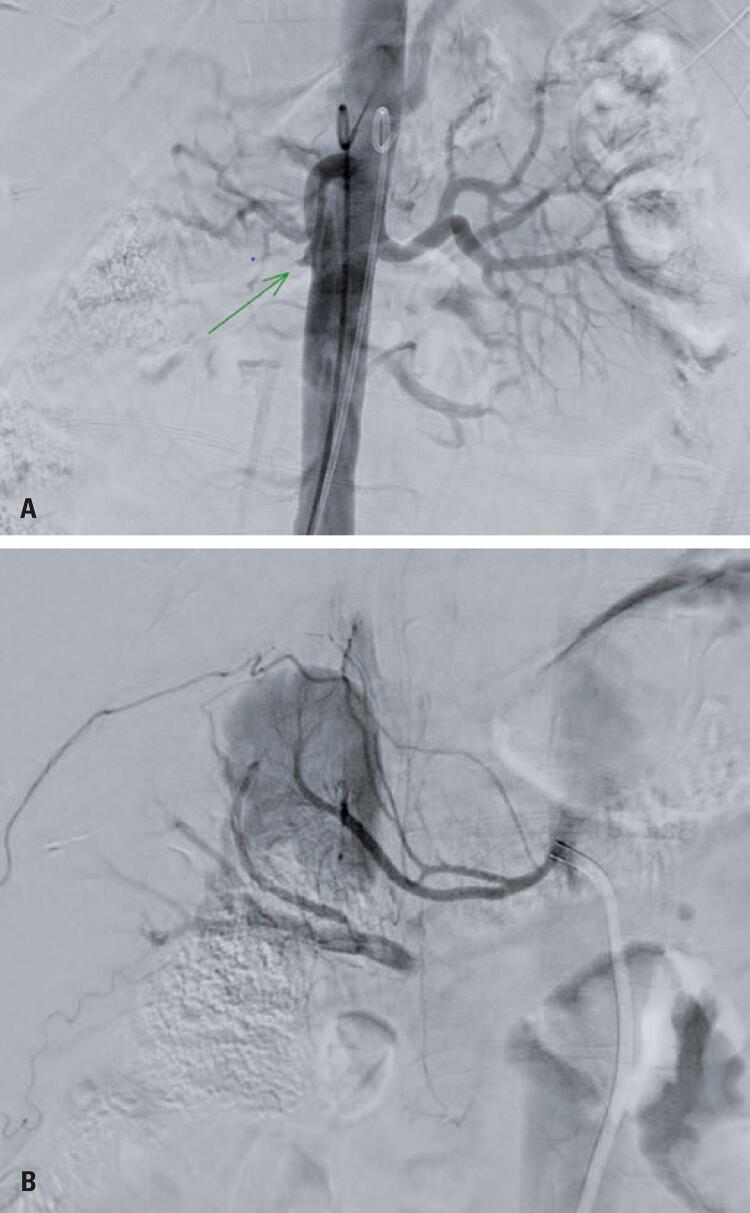
(A) Digital subtraction angiography. Right renal artery stump. (B) Digital subtraction angiography. Polar renal artery catheterization with retrograde filling of the right renal artery

This was followed by a digital subtraction angiography of the renal arteries, to complement diagnostic and therapeutic assessments. Aortography revealed occlusion of the right renal artery, in addition to identification of an upper renal polar artery, whose selective catheterization allowed retrograde contrasting of the main renal artery, showing a double lumen associated with an intraluminal thrombus in the right renal artery, suggesting dissection (Figures 2A and 2B).

**Figure 3:**
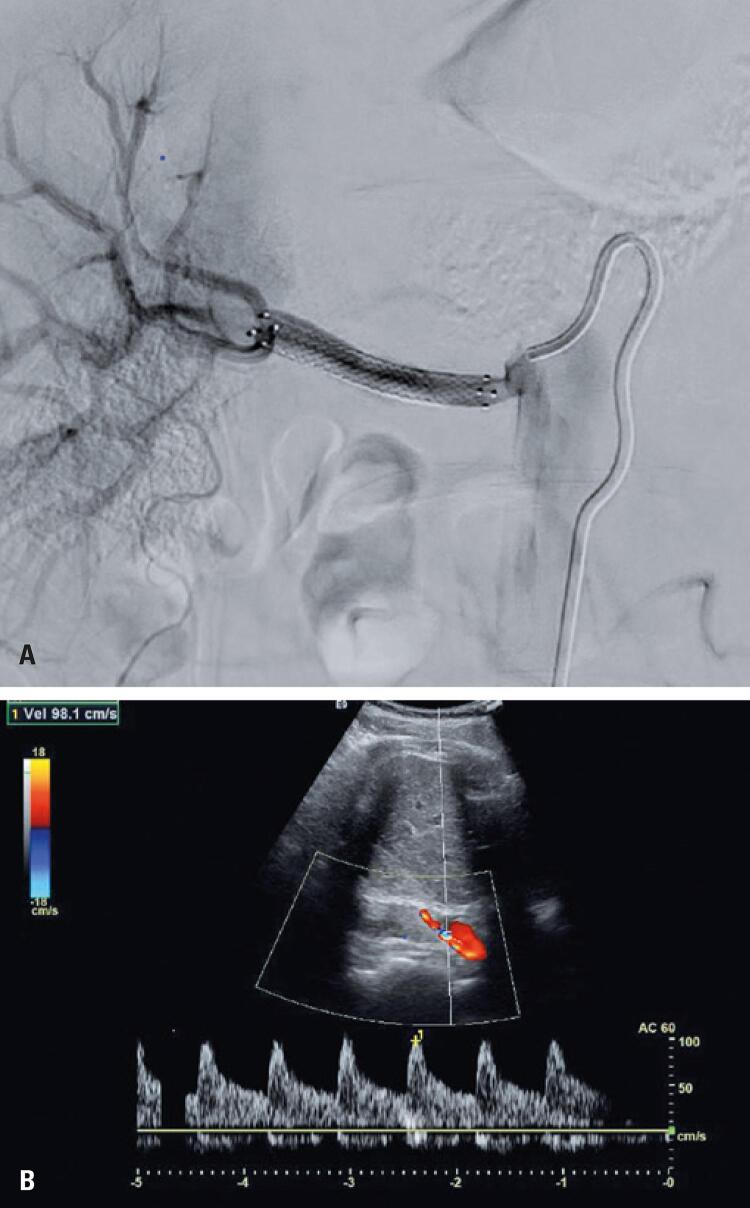
(A) Post-procedure control. Digital subtraction angiography. Final control after the first procedure, showing a patent and well-positioned stent and adequate perfusion of intrarenal branches. (B) Post-procedure control. Digital subtraction angiography. Doppler ultrasound showing reduced peak systolic velocity in the right renal artery

Therefore, thrombus aspiration was chosen, successfully using a Penumbra System^®^ device, followed by renal artery angioplasty with an Emerge™ 4mm x 30mm balloon (Boston Scientific) and a Zilver^®^ 4mm x 40mm stent implantation (Cook Medical). Due to the poor positioning of the first stent, we chose to deploy a second balloon-expandable stent, for better precision in the coverage of the renal artery ostium with a Dynamic^®^ 5mm x 20mm stent (Biotronik). A control arteriography showed good stent placement with maintenance of renal artery patency, and good contrasting of distal branches, renal parenchyma and venous phase (Figure 3A).

In the immediate postoperative period, a new Doppler ultrasound of the right renal artery showed an improvement in the peak systolic velocity (approximately 140cm/s) and intrarenal resistive indices of roughly 0.80 (Figure 3B). In less than 24 hours, the patient was weaned from intravenous antihypertensive agents, with satisfactory pressure control (123mmHg x 77mmHg) and normal creatinine levels (1.14).

**Figure 4:**
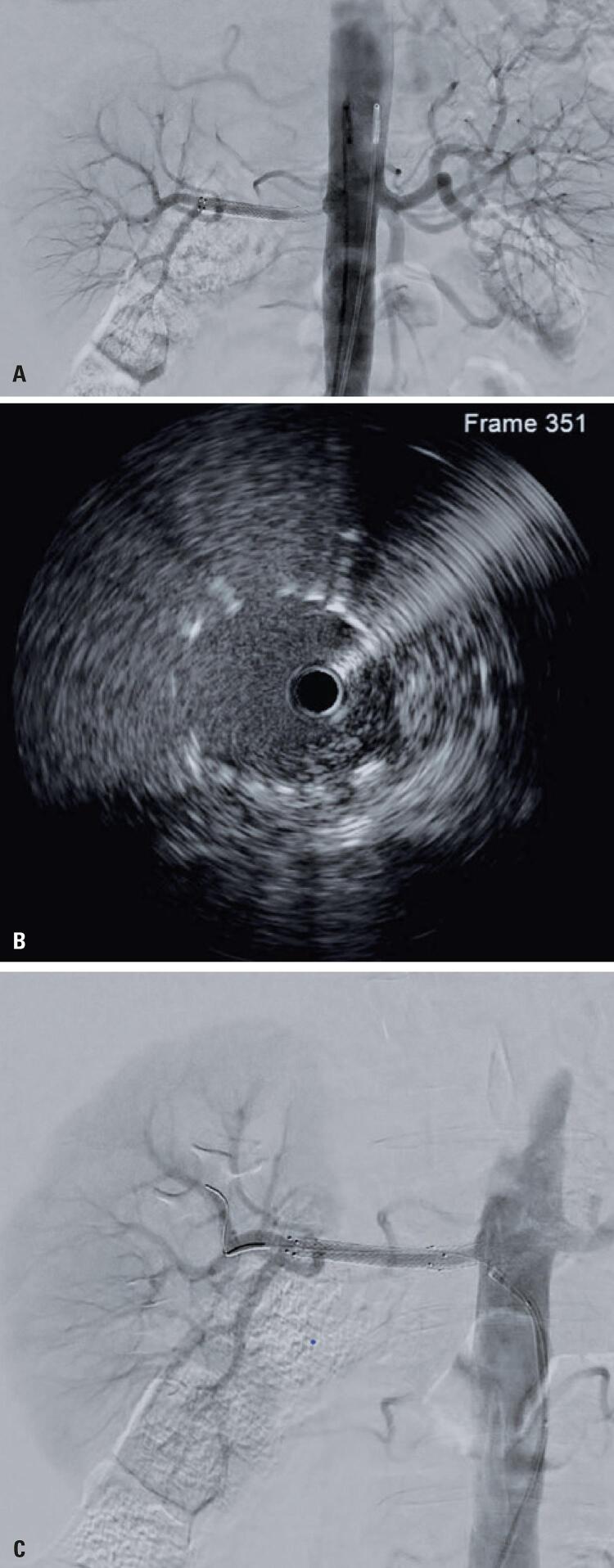
(A) Diagnostic arteriography of the second intervention, showing luminal reduction in the proximal third of the right renal artery, extending to the stent, suggestive of intimal hyperplasia. (B) Intravascular ultrasound showing adequate stent placement. (C) Post-angioplasty arteriography of the second procedure showing patency of the right renal artery

After a 6-month follow-up and appropriate hypertensive control with amlodipine, the patient presented with a new increase in blood pressure. A new ultrasonographic evaluation confirmed increased in-stent systolic velocity peaks (greater than 450cm/s) with low intrarenal flow. Therefore, a new angiography was performed. The angiographic evaluation showed restenosis in the proximal portion of the stent (Figure 4A), which was more thoroughly evaluated with intravascular ultrasonography (Philips) (Figure 4B) and treated with balloon angioplasty. The evaluation with intravascular ultrasound after ballooning identified an adequate stent placement and improved lumen patency. This was confirmed by a control angiography (Figure 4C) and an improvement in pressure parameters obtained with a Doppler ultrasonography performed in the first postoperative period. The patient maintained good control of blood pressure parameters after nearly 6 months of follow-up after the second interventional procedure.

This study was approved by the Research Ethics Committee of *Hospital Israelita Albert Einstein* under # 4.186.693, CAAE: 35449420.9.0000.0071.

## DISCUSSION

Spontaneous renal artery dissection was first described by Bumpus, in 1944, and is defined as a dissection unrelated to previous trauma or arterial interventions.^(3)^ This disease is a rare cause of renal artery stenosis, with an incidence and prevalence difficult to assess, notably due to the non-specificity of the clinical manifestations, but with a clear predominance, not yet elucidated, in men (4:1).

Symptoms are nonspecific and include both acute and chronic presentations. Acute symptoms such as low back pain, resistant hypertension, hematuria, fever, leukocytosis, among others, are described, although most patients are initially asymptomatic.^(4-6)^ In chronic cases, complaints related to difficult-to-control hypertension are reported and gastrointestinal and urinary symptoms that can mimic other diseases, making the diagnosis difficult.^(7)^ The rarity of the disease and the non-specificity of symptoms highlight the importance of identifying risk factors, such as fibromuscular dysplasia, strenuous exercise, use of cocaine, among others, for quickest investigation of this disease. In this case report, the patient had a history of intense physical exercise in the previous days, which may have been a triggering factor for the condition.

Among the diagnostic methods, ultrasound, computed tomography angiography and magnetic resonance angiography are classical methods, with good sensitivity and specificity.^(8)^ Other diagnostic methods that can be used are intravascular ultrasonography and optical coherence tomography (OCT), which provide a detailed assessment of the vessel wall.^(7)^ In case of high clinical suspicion and impairment of renal function, the investigation should be performed initially with ultrasonography,^(8)^ followed by arteriography, in doubtful cases or with a therapeutic objective.

The initial treatment usually includes clinical management with antihypertensive and symptomatic drugs, in addition to anticoagulation. In cases that are resistant to this initial approach, there are no clear guidelines on management.^(4)^ In this scenario, interventional and surgical treatments are options. Surgical management includes vascular restorative therapy or nephrectomy in more advanced cases. The endovascular approach includes balloon angioplasty, embolization, and stenting. The endovascular treatment is indicated in symptomatic patients, with residual functional reserve and favorable anatomy,^(4)^ after failure or intolerance to drug treatment, who have resistant hypertension, with loss of renal function or progressive congestive heart failure.^(9-12)^

With regard to endovascular treatment, the 2015 American College of Radiology/Society of Interventional Radiology (ACR-SIR) document recommends the use of stents in the case of dissection, with a moderate level of evidence (2B).^(13)^ Short-segment focal dissections with more than 50% stenosis are more amenable to repair, by reopening the arterial lumen and stabilizing the intimal flap.^(4)^

Unlike what happens in the treatment of atherosclerotic lesions, the long-term follow-up of angioplasty using stent in dissection cases shows low rates, or even absence of restenosis.^(2)^ This is a relevant difference between what is expected in the literature and what was observed in this case report, since the patient experienced symptomatic stenosis within a short post-treatment period (6 months).

## CONCLUSION

Spontaneous renal artery dissection is a rare cause of renal artery stenosis, which can lead to a severe hypertension that is difficult to control, and there is still no consensus on the ideal treatment in drug-resistant cases. In this scenario, the minimally invasive endovascular approach had good results, and should be considered among the therapeutic options.
